# The *Ph-3* gene from *Solanum pimpinellifolium* encodes CC-NBS-LRR protein conferring resistance to *Phytophthora infestans*

**DOI:** 10.1007/s00122-014-2303-1

**Published:** 2014-04-23

**Authors:** Chunzhi Zhang, Lei Liu, Xiaoxuan Wang, Jack Vossen, Guangcun Li, Tao Li, Zheng Zheng, Jianchang Gao, Yanmei Guo, Richard G. F. Visser, Junming Li, Yuling Bai, Yongchen Du

**Affiliations:** 1 The Institute of Vegetables and Flowers, Chinese Academy of Agricultural Sciences, Zhongguancunnandajie 12, 100081 Beijing, People’s Republic of China; 2 Wageningen UR Plant Breeding, Wageningen University and Research Center, Droevendaalsesteeg 1, 6708 PB Wageningen, The Netherlands; 3 Institute of Vegetables and Flowers, Shandong Academy of Agricultural Sciences, 250100 Jinan, People’s Republic of China

## Abstract

*****Key message***:**

***Ph-3*** **is the first cloned tomato gene for resistance to late blight and encodes a CC-NBS-LRR protein.**

**Abstract:**

Late blight, caused by *Phytophthora infestans*, is one of the most destructive diseases in tomato. The resistance (*R*) gene *Ph*-*3*, derived from *Solanum pimpinellifolium* L3708, provides resistance to multiple *P. infestans* isolates and has been widely used in tomato breeding programmes. In our previous study, *Ph*-*3* was mapped into a region harbouring *R* gene analogues (RGA) at the distal part of long arm of chromosome 9. To further narrow down the *Ph*-*3* interval, more recombinants were identified using the flanking markers G2-4 and M8-2, which defined the *Ph*-*3* gene to a 26 kb region according to the Heinz1706 reference genome. To clone the *Ph*-*3* gene, a bacterial artificial chromosome (BAC) library was constructed using L3708 and one BAC clone B25E21 containing the *Ph*-*3* region was identified. The sequence of the BAC clone B25E21 showed that only one RGA was present in the target region. A subsequent complementation analysis demonstrated that this RGA, encoding a CC-NBS-LRR protein, was able to complement the susceptible phenotype in cultivar Moneymaker. Thus this RGA was considered the *Ph*-*3* gene. The predicted Ph-3 protein shares high amino acid identity with the chromosome-9-derived potato resistance proteins against *P. infestans* (Rpi proteins).

**Electronic supplementary material:**

The online version of this article (doi:10.1007/s00122-014-2303-1) contains supplementary material, which is available to authorized users.

## Introduction

Late blight, caused by *Phytophthora infestans*, is one of the most devastating diseases for field-grown tomatoes. Under favourable conditions, *P. infestans* can spread at an alarming pace, and the compatible host will be devastated within 7–10 days (Fry 2008). Fungicide treatment is currently the most common method to control late blight. However, fungicide application is costly and has a negative impact on human health and environmental safety. Moreover, the pathogen quickly evolves and some of the new variants are insensitive to commonly used fungicides (Goodwin et al. 1996). The disease is especially problematic for organic growers who do not use any chemical pesticides in the production process. Therefore, introduction of resistances from wild tomato species into cultivated tomato is considered as a valuable method to achieve durable late blight resistance.

Currently, more than 60 *Solanum* resistance genes against *P. infestans* (*Rpi* genes), mainly in potato, have been located in 16 regions on 10 chromosomes (Rodewald and Trognitz 2013). Among them, some have been cloned through map-based cloning or allele mining (Rodewald and Trognitz 2013). Additional information on characterization of these cloned *Rpi* genes in potato can be found in numerous recent reviews related to this topic (Foolad et al. 2008; Hein et al. 2009; Vleeshouwers et al. 2011; Nowicki et al. 2012; Rodewald and Trognitz 2013). Most of the *Rpi* genes are identified in wild potatoes, such as *S. demissum*, *S. bulbocastanum*, *S. venturii*, etc. (Vleeshouwers et al. 2011; Rodewald and Trognitz 2013). In tomato, much less studies on late blight resistance have been carried out. This is in part because this pathogen in tomato was not as prevalent as in potato, at least before the 1990s when many of the potato isolates were not pathogenic to tomato (Nowicki et al. 2012). However, tomato *P. infestans* isolates have recently undergone significant genetic changes and are becoming one of the most devastating pathogens for tomato cultivation (Foolad et al. 2008).

*P. infestans* is heterothallic, and both A1 and A2 mating types are required for completion of the sexual cycle. Sexual reproduction results in high levels of genetic variation in the offspring and may lead to rapid pathogen evolution and thus increases the risk of epidemics (Foolad et al. 2008). In the latest reports, tomato *P. infestans* isolates collected in China and Tunisia are still A1 mating types (Guo et al. 2010; Li et al. 2013; Harbaoui et al. 2013). In the USA, however, the predominant clonal lineage US-22 in Wisconsin is A2 mating type and resulted in the epidemics on tomato in 2009 (Gevens and Seidl 2013). The A2 mating type of tomato *P. infestans* isolates has also been reported in Russia (Statsyuk et al. 2010). In South-West India, appearance of the (blue) 13_A2 lineage caused severe outbreaks of late blight on tomatoes from 2009 to 2010 (Chowdappa et al. 2013). In potato cultivation, the aggressive 13_A2 lineage has emerged in Northwest Europe and rapidly replaced other genotypes (Cooke et al. 2012). This lineage is also present in the population of potato *P. infestans* in China (Li et al. 2013), but has not been collected yet in the Chinese tomato *P. infestans* population.

Due to the recent increased significance of tomato late blight, more effort is needed to identify genetic resources for late blight resistance and transfer the resistance to breeding lines and cultivars. To date, resistance to *P. infestans* has been reported in wild tomato species. The *Ph*-*1* gene is the first reported *Rpi* gene in tomato, which is a dominant gene mapped on chromosome 7 and provides resistance against *P. infestans* isolate T_0_. The *Ph*-*1* gene was originally identified in *Solanum pimpinellifolium* accessions known as West Virginia 19 and 731 and has been introduced into the cultivated tomato (Bonde and Murphy 1952; Gallegly and Marvel 1955; Rich et al. 1962; Peirce 1971). The second *Rpi* gene *Ph*-*2* was identified in another *S. pimpinellifolium* accession (West Virginia 700) (Gallegly and Marvel 1955). The *Ph*-*2* gene, conferring incomplete late blight resistance, was mapped into an 8.4-cM interval on the long arm of chromosome 10 (Moreau et al. 1998). This gene provides partial resistance resulting in only a reduction in the rate of disease development (Goodwin et al. 1995; Black et al. 1996a). Resistance conferred by both *Ph*-*1* and *Ph*-*2* was overcome by different *P. infestans* isolates from China, Indonesia, Nepal and The Philippines (AVRDC 1995, 1998, 1999), which prompted further screening of tomato germplasm for new *Rpi* genes. As a result, *S. pimpinellifolium* L3708 was found to be highly resistant to a wide range of *P. infestans* isolates overcoming *Ph*-*1* and *Ph*-*2* (Black et al. 1996a, b). The late blight resistance in L3708 is conditioned by a partially dominant gene, *Ph*-*3*, which was mapped on the long arm of chromosome 9 (Black et al. 1996a; Chunwongse et al. 2002; Zhang et al. 2013). With marker-assisted selection (MAS) using *Ph*-*3*-linked molecular markers, this gene has been successfully introgressed into tomato breeding lines and tomato cultivars for both commercial processing and fresh-market (Foolad et al. 2008; Gardner and Panthee 2010a, b; Panthee and Gardner 2010; Robbins et al. 2010). However, the resistance conferred by *Ph*-*3* is also race-specific, and the isolates virulent on L3708 have already been identified (Chunwongse et al. 2002). Another reported late blight resistant accession is *S. habrochaites* LA1033, which was designated as the source of *Ph*-*4* (AVRDC 1998). LA1033 was used as one of the differential hosts to classify tomato *P. infestans* isolates (Kim and Mutschler 2000; Chunwongse et al. 2002). Characterization of *Ph*-*4* has been hampered because follow-up investigations revealed that the resistance in LA1033 was actually controlled by multiple quantitative trait loci (QTLs) (Lough 2003; Kim and Mutschler 2000). Recently, a new resistant line, *S. pimpinellifolium* PSLP153, has been discovered which showed resistance against seven different *P. infestans* isolates (Foolad et al. 2006, 2008). Two genomic regions on chromosome 1 (tentatively named *Ph*-*5*-*1*) and chromosome 10 (tentatively named *Ph*-*5*-*2*) were identified through a selective genotyping approach (Merk et al. 2012; Merk and Foolad 2012; Nowicki et al. 2012). Efforts are underway to develop commercial breeding lines and hybrid cultivars containing these resistance genes in combination with *Ph*-*2* and *Ph*-*3* (Foolad et al. 2008; Nowicki et al. 2012).

Other QTLs conferring race-non-specific resistance have been identified from *S. pennellii* and *S.* *habrochaites* (Smart et al. 2007; Brouwer et al. 2004; Brouwer and St. Clair 2004; Li et al. 2011a). However, the effects of these QTLs are relatively small and prone to environmental influences. Moreover, linkage drag might complicate the use of these QTLs in breeding programmes (Brouwer and St. Clair 2004).

Currently, introgression or pyramiding of *R* genes via traditional breeding may not always be possible or too time-consuming. An alternative approach to introduce single or multiple *R* genes is genetic transformation (Halpin 2005). To achieve durable resistance, three potato *Rpi* genes were introduced into one genotype through a one-step transformation strategy, and the resulting plants showed an expected broadened resistance spectrum (Zhu et al. 2012, 2013). This approach, however, requires prior knowledge of the gene(s), including mapping, cloning, and functional characterization.

In this study, we performed map-based cloning to isolate *Ph*-*3*, a gene encoding a coiled-coil nucleotide-binding leucine-rich repeat (CC-NBS-LRR) protein. Further, we analysed the Ph-3 protein structure and compared it with other Rpi proteins characterized so far from potato.

## Materials and methods

### Plant materials

The *S. lycopersicum* accessions CLN2037B and CLN2037E, containing the *Rpi* gene *Ph*-*3*, were kindly provided by the Asian Vegetable Research and Development Center (AVRDC). These two cultivars were crossed with the susceptible tomato breeding line 02393, respectively. Recombinant screening was conducted using the F_2_ seeds with *Ph*-*3* flanking markers G2-4 and M8-2 (Table 1). In addition, eight F_3_ families (B212, B481, N299, N337, N1036, N1097, N1200, N1384), which were derived from the cross between CLN2037B and LA4084 (susceptible) and identified in our previous study (Zhang et al. 2013), were also used for screening recombinants (about 150 plants per F_3_ family) with the same markers.

**Table 1:** Markers used for recombinant screening

Marker names	Forward primer (5′–3′)	Reverse primer (5′–3′)	Type of marker
G2-4	ATGCCACGACCATAAATC	GACTGGGCTAATCACGAA	CAPS with *Dra*I
R1-3U	AAAAGTATTCAGAGGGGTAA	ATTGCAGATCCATTTCAGT	Co-dominant SCAR
R2-3U	TAGTGACACGCTGATAAC	CAATTCTTTGTTGGAGAC	dominant
R2M1S	GGAAATCCTCCGCCTTACTT	CGAGTTGCAACCTCTAGACTCA	Co-dominant SCAR
TG591S	GCGAGACATAGACCAATC	AACTGGCAGGTGATGTGG	SNP
M67-3	TGCGAATCCTTGTGGTAT	CTTACTGTGGACTGTGGG	CAPS with *Ssp*I
G7-5	TGCCTCTGTGAAGATGGT	AAACTGTCGCAGGGTATT	SNP
G8-1	CGCCGTTTCGTGGCATTT	AGCGTGGTGATGGTGTTT	SNP
M8-2	AGGTGTCTCATTCCCATCA	ATAGGGACCAATAGAGGG	InDel

(Co-)dominant indicates that it is a (Co-)dominant marker. InDel indicates that this marker is derived from a short Insert/Deletion variation

*CAPS* cleaved amplified polymorphic sequence, *SCAR* sequence-characterized amplified region, *SNP* single-nucleotide polymorphism

### Marker development

According to our previous work (Zhang et al. 2013), the target region of *Ph*-*3* on the Heinz1706 reference genome (http://solgenomics.net) was selected to design PCR primers. Amplified PCR products from the parental lines were sequenced and analysed for polymorphisms in order to produce cleaved amplified polymorphic sequences (CAPS) or insert/deletion (InDel) markers.

### Construction and screening of BAC library

The bacterial artificial chromosome (BAC) library was generated using the *Ph*-*3* donor species *S. pimpinellifolium* L3708 with restriction enzymes *Hind*III according to the previously described protocol (van der Voort et al. 1999). The BAC library was stored in 252 384-well microtiter plates, and all 384 clones in one plate were mixed to form a BAC pool. The BAC pool DNA was isolated by alkaline lysis method and screened with two markers TG591S and R2M1S that are closely linked to *Ph*-*3*. Afterwards, the single colony from the 384-well plates corresponding to the positive pool was identified using the same markers. DNA from the single positive colony was isolated and then tested with additional markers covering the *Ph*-*3* region (Table 1).

### DNA sequencing and analysis

Sequence of the selected BAC clone harbouring the *Ph*-*3* region was obtained by constructing a library of subclones (1–3 kb). Both ends of the subclones were sequenced using the ABI 3730xl platform and then assembled (BGI, Beijing, China). Putative genes in the BAC sequence were predicted with the online program FGENESH (http://linux1.softberry.com/) and protein functions were predicted with the InterProScan program (http://ebi.ac.uk/Tools/InterProScan/). Results were compared with the Heinz1706 genome annotations derived from the International Tomato Annotation Group (ITAG2.3 version). ClustalW2 was used to align multiple sequences with default settings (http://www.ebi.ac.uk/Tools/msa/clustalw2/).

### Transformation of the *Ph*-*3* gene into the susceptible *S. lycopersicum* cv. Moneymaker

A 8-kb fragment carrying the *Ph*-*3* promoter, open reading frame (ORF) and terminator was amplified from the BAC plasmid B25E21 by PCR using the Phusion high-fidelity DNA polymerase (Thermo Fisher, Waltham, MA, USA) with primers Ph3EF3 (5′-taa*cctgcagg*TTCAAACCATCTTCATAGAGGC-3′) and Ph3ER3 (5′-att*ggcgcgcc*TGGGGCTTAGAAAAAGGTTG-3′). Two enzyme sites *Sbf*I and *Asc*I were added to the 5′ ends of forward and reverse primers, respectively. The PCR product was cloned into pCR-Blunt II-TOPO (Invitrogen, Carlsbad, CA, USA) and sequenced for confirmation. The resulting plasmid was digested with *Sbf*I and *Asc*I. The fragment containing the *Ph*-*3* gene was then ligated into the binary vector pBINPLUS having a modified multiple cloning site. The positive plasmid, named Ph13-2, was introduced into *Agrobacterium tumefaciens* strain AGL1 by electroporation.

Transformation of *S. lycopersicum* cv. Moneymaker was carried out as described by Huibers et al. (2013). Twenty-four regenerants that were capable of growing on kanamycin medium were transferred to the greenhouse. All kanamycin resistant regenerants were screened with the primer pair M67-3F (5′-TGCGAATCCTTGTGGTAT-3′, located in the *Ph*-*3* fragment) and pBP-R2 (5′-AGGGAAGAAAGCGAAAGGAG-3′, located in the vector but within the T-DNA region).

### Disease assay

Both whole-plant assay (WPA) and detached-leaf assay (DLA) were used for disease tests with *P. infestans*. The progenies of two recombinants (1-356 and 8-25) were tested by WPA as described by Zhang et al. (2013). The recombinants and *Ph*-*3* transgenic plants were tested for *P. infestans* resistance through DLA as described by Vleeshouwers (1999). Three leaves of each plant were used and inoculated with *P. infestans* isolate T_1,2,4_ (Zhang et al. 2013). Two independent disease tests were performed for DLA.

### RNA isolation and quantitative real-time PCR

Total RNA was extracted using RNeasy plant mini kit (Qiagen, Hilden, Germany). First-strand cDNA was synthesized with the iScript cDNA synthesis kit (Bio-Rad, Hercules, CA, USA). cDNA was diluted tenfold and used for real-time PCR (RT-PCR) with the Bio-Rad CFX96™ thermal cycler according to the protocol provided by the manufacturer. To detect the expression of *R* gene analogues (RGAs) in CLN2037B, samples were taken from three plants, and for each plant three leaves were pooled for RT-PCR. The primers R1eF1 (5′-GAAAGGGATGCAAGAACCAA-3′) and R1eR1 (5′-CGACAAACTTGTTGGCAGAA-3′) located in ORF2, which produced a 181-bp fragment, were used to test the expression of ORF2. The primers used to check the expression of ORF3 were R2eF1 (5′-TTCTTCTTACTGCAGTCGTCAA-3′) and R2eR1 (5′-TCCAACTTCCTTTGCCTTTG-3′), which produced a 164-bp fragment. For analysis of the *Ph*-*3* expression level in the primary transgenic plants, the primers R2eF1 and R2eR1 were used. The tomato *elongation factor 1α* (EF1α) gene (Gene ID: 544055) was used as the internal reference in all analyses which was amplified with forward (5′-ATTGGAAACGGATATGCTCCA-3′) and reverse primers (5′-TCCTTACCTGAACGCCTGTCA-3′). Gene expression level was calculated on the basis of the 2^−∆∆Ct^ method (Livak and Schmittgen 2001).

## Results

### Fine mapping of the *Ph*-*3* gene

Previously, *Ph*-*3* was mapped into a 74-kb interval on the long arm of chromosome 9 (Zhang et al. 2013). In this region, eight genes were identified in the Heinz1706 reference genome (The Tomato Genome Consortium 2012). To further narrow down the *Ph*-3 interval, approximately 1,900 plants from two F_2_ populations (CLN2037B × 02393 and CLN2037E × 02393) and eight F_3_ populations (derived from CLN2037B × LA4084) were screened with two markers G2-4 and M8-2 flanking the *Ph*-*3* gene (Table 1). Seven recombinants were identified and genotyped with additional markers located in between G2-4 and M8-2. Three leaves of each recombinant were inoculated with *P. infestans* isolate T_1,2,4_ through DLA. In two independent experiments, five recombinants (1-104, 4-35, 1-356, 7-111, 4-54) containing the *S. pimpinellifolium* L3708 introgression between markers G2-4 and M67-3 were resistant, while two recombinants (8-25 and 2-125) lacking this introgression were susceptible (Table 2). This result indicated that *Ph*-*3* was located in between markers G2-4 and M67-3, a region of 41 kb in the Heinz1706 genome. The progenies of two important recombinants (1-356 and 8-25) were tested with *P. infestans* using the whole-plant assay. In the progeny of 1-356, two out of the 10 tested plants were susceptible, suggesting that the *Ph*-*3* gene was located in the heterozygous region, upstream of the marker M67-3. All 27 progeny plants from 8–25 were susceptible confirming that the introgression between markers G2-4 and R2M1S did not carry the late blight resistance. Therefore, the *Ph*-*3* gene was delimited to a 26 kb region between markers R2M1S and M67-3 based on the Heinz1706 reference genome.

**Table 2:** Phenotype and genotype of the identified recombinants

Recombinants	Populations	Marker name and genotype									Phenotype
		G2-4	R1-3U	R2-3U	R2M1S	TG591S	M67-3	G7-5	G8-1	M8-2	
1-104	F_2_ of CLN2037B × 02393	b	h	h	h	h	h	h	h	h	R
4-35	F_3_ of B481 from CLN2037B × LA4084	b	h	h	h	h	h	h	h	h	R
8-25	F_3_ of N337 from CLN2037B × LA4084	h	h	h	h	a	a	a	a	a	S
1-356	F_2_ of CLN2037B × 02393	h	h	h	h	h	b	b	b	b	R
7-111	F_3_ of B212 from CLN2037B × LA4084	h	h	h	h	h	h	a	a	a	R
2-125	F_2_ of CLN2037E × 02393	a	a	a	a	a	a	a	/	h	S
4-54	F_3_ of B481 from CLN2037B × LA4084	b	b	b	b	b	b	b	b	h	R

*a*, homozygous like the susceptible parent; *b*, homozygous like the resistant parent; *h*, heterozygous; /, not determined

### BAC library screening

In the previous study, we have demonstrated that the *Ph*-*3* gene belongs to a CC-NBS-LRR gene family at the end of the long arm of chromosome 9. In the Heinz1706 genome, there are four members of this gene family present in the 74-kb interval where *Ph*-*3* is located (Zhang et al. 2013). We thus tried to amplify the full length of the homologous alleles in *S. pimpinellifolium* L3708 (the donor of the *Ph*-*3* gene). Unfortunately, primers designed according to the Heinz1706 reference genome failed to amplify the full length of candidate homologs from the *Ph*-*3*-carrying tomato lines. In order to obtain the sequence covering the *Ph*-*3* locus, the *Ph*-*3* donor *S. pimpinellifolium* L3708 was used to construct a BAC library. The library consisted of 96,768 clones with an average insert size of 100 kb based on pulsed-field gel analysis of randomly selected clones. The library was thus estimated to represent approximately tenfold coverage of the L3708 genome.

**Fig. 1:**
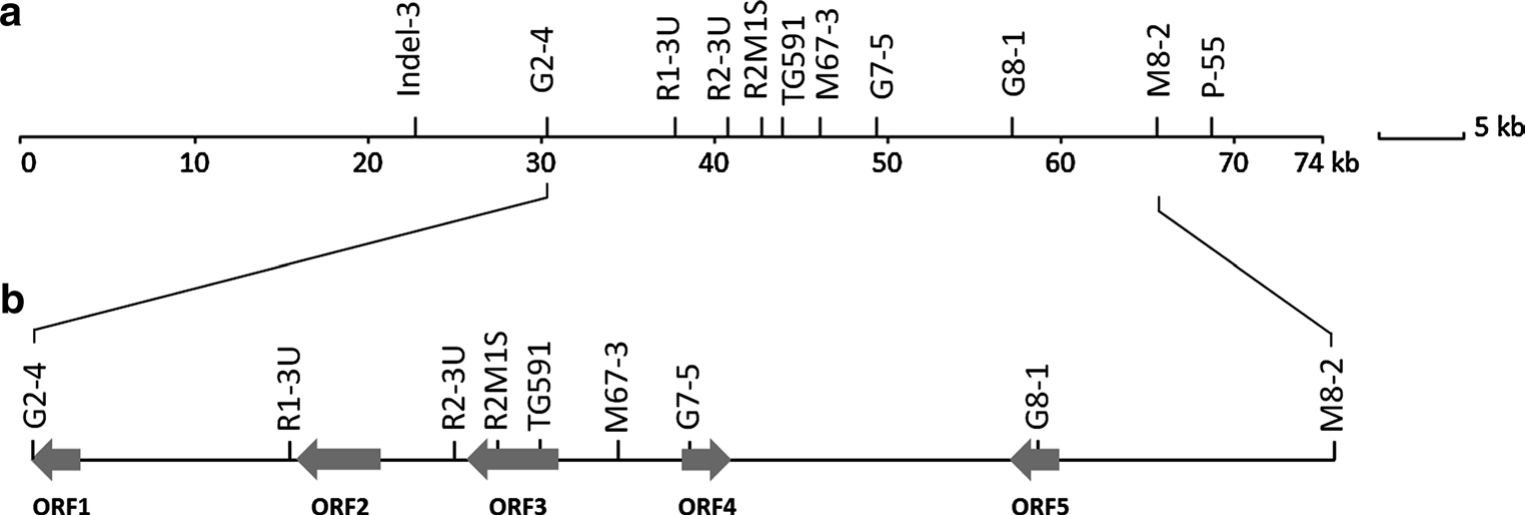
Physical map of the *Ph*-*3* genomic region from *S. pimpinellifolium* L3708. **a** Positions of markers in BAC clone B25E21. Indel-3 and P-55 were previously identified *Ph*-*3* flanking markers (Zhang et al. 2013). **b** The predicted ORFs between markers G2-4 and M8-2

**Fig. 2:**
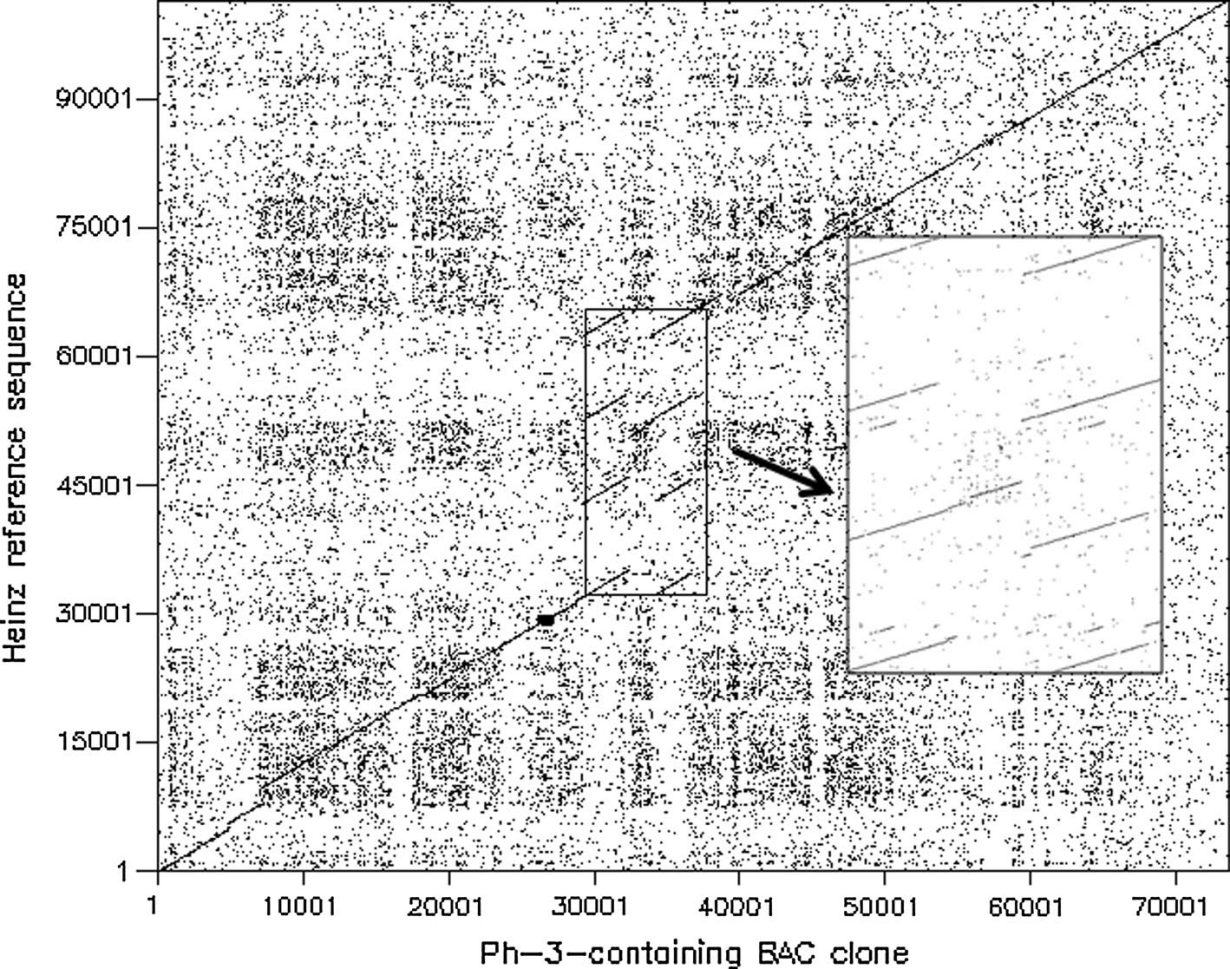
Comparison of the Ph-3-containing BAC sequence with the Heinz1706 reference sequence. The *X*-axis shows the sequence of BAC B25E21, and the *Y*-axis shows the corresponding Heinz1706 reference sequence. The sequences were analysed using dottup (v6.0.1) with a window size 10. *Arrow* points to the large picture of the variable region carrying RGAs

Two PCR markers R2M1S and TG591S (Fig. 1), which were closely linked to *Ph*-*3,* were used to screen the BAC library. A positive BAC pool B25 was identified from which a positive clone B25E21 was picked up. Subsequently, the full length of B25E21 was sequenced. The whole BAC sequence of B25E21 carries an insert of 73,671 bp from *S. pimpinellifolium* L3708, corresponding to an interval of 101,456 bp in the Heinz1706 reference genome starting from SL2.40ch09:66725592 and ending at SL2.40ch09:66827013. The sequence alignment showed that the first 29 kb and the last 37 kb of BAC clone B25E21 were collinear with the reference sequence except two short deletions in the first 10 kb (Fig. 2). The major difference was the high variable region in the middle, starting at about 66,762 kb–66,795 bp based on the Heinz1706 genome.

All markers used for screening recombinants (Table 1) were found in the BAC sequence (Fig. 1a). In total, five ORFs between the *Ph*-*3* flanking markers G2-4 and M8-2 were predicted. Among them, ORF1 encodes a transferase, ORF2 and ORF3 are *RGAs* encoding CC-NBS-LRR type of R proteins, ORF4 encodes an RNA binding protein-like protein and ORF5 encodes an NAD-dependent epimerase (Fig. 1b).

### Candidate of *Ph*-*3*

**Fig. 3:**
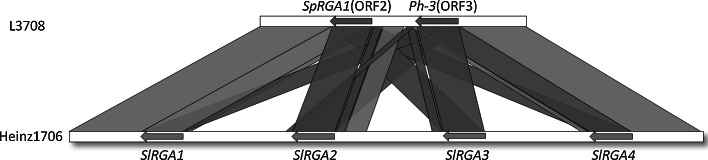
Schematic of the microsynteny between the *R* gene clusters at the *Ph*-*3* locus in *S. pimpinellifolium* L3708 and *S. lycopersicum* Heinz1706. The *green arrows* at the *top* show the *R* gene homologs in the L3708 BAC sequence, and the *yellow arrows* at the *bottom* indicate RGAs at the corresponding locus of Heinz1706. The transcriptional orientations are indicated by the direction of *arrows*. The *orange, purple and blue lines* linking the L3708 and Heinz1706 sequences indicate an identity above 95, 90–95, and 85–90 %, respectively

In the previous study, we have demonstrated that *Ph*-*3* is an RGA of an NBS-type family (Zhang et al. 2013). In the Heinz1706 genome, the *Ph*-*3* interval carries four RGA members (*SlRGA1*-*SlRGA4*). However, in *S. pimpinellifolium* L3708 genome, it contains only two RGAs (ORF2 and ORF3) (Fig. 3), which show different nucleotide identity to the four RGAs in the Heinz1706 genome (Fig. 3; Table S1). In the tomato line CLN2037B carrying the *Ph*-*3* gene, the expression of ORF3 but not ORF2 was detected (Fig. S1). Since *Ph*-*3* was mapped to a 26 kb interval between markers R2M1S and M67-3 (Table 2), a region harbouring only ORF3, the ORF2 was thus excluded to be the *Ph*-*3* candidate.

**Fig. 4:**
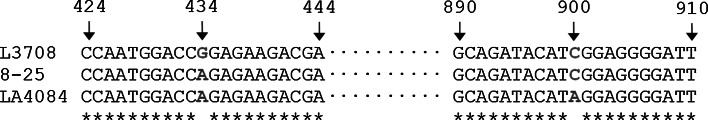
The recombination point in the key recombinant 8-25. The ORF3 fragments from the *Ph*-*3* donor plant L3708, the susceptible parent LA4084 and the susceptible recombinant 8-25 were aligned. Based on two SNPs (residues 434 and 900) in this region, the recombination site of 8-25 was located within the ORF3. The numbers above the *arrows* indicate the positions of nucleotides in the ORF3

The key recombinant 8-25 which resulted from recombination events in the RGAs region was analysed. This susceptible recombinant is heterozygous at the R2M1S locus while it is homozygous for the LA4084 allele at the TG591S locus. Both R2M1S and TG591S are located within ORF3. The progeny plants of the recombinant 8-25, which was homozygous for the CLN2037B allele at R2M1S locus and homozygous for the LA4084 allele at TG591S locus were selected and used to amplify the DNA fragment with the R2M1S forward primer and the TG591S reverse primer. Subsequently, this sequence was aligned with the alleles from *S. pimpinellifolium* L3708 and the susceptible parent LA4084. In this way, the crossing-over event of 8-25 was pinpointed between two SNPs (Fig. 4) which are 465 bp apart. Since all progeny plants of 8-25 were susceptible, it is very likely that the recombination event in 8-25 led to a non-functional chimeric ORF3, suggesting that ORF3 was the most likely candidate of *Ph*-*3*.

### Complementation analysis

**Fig. 5:**
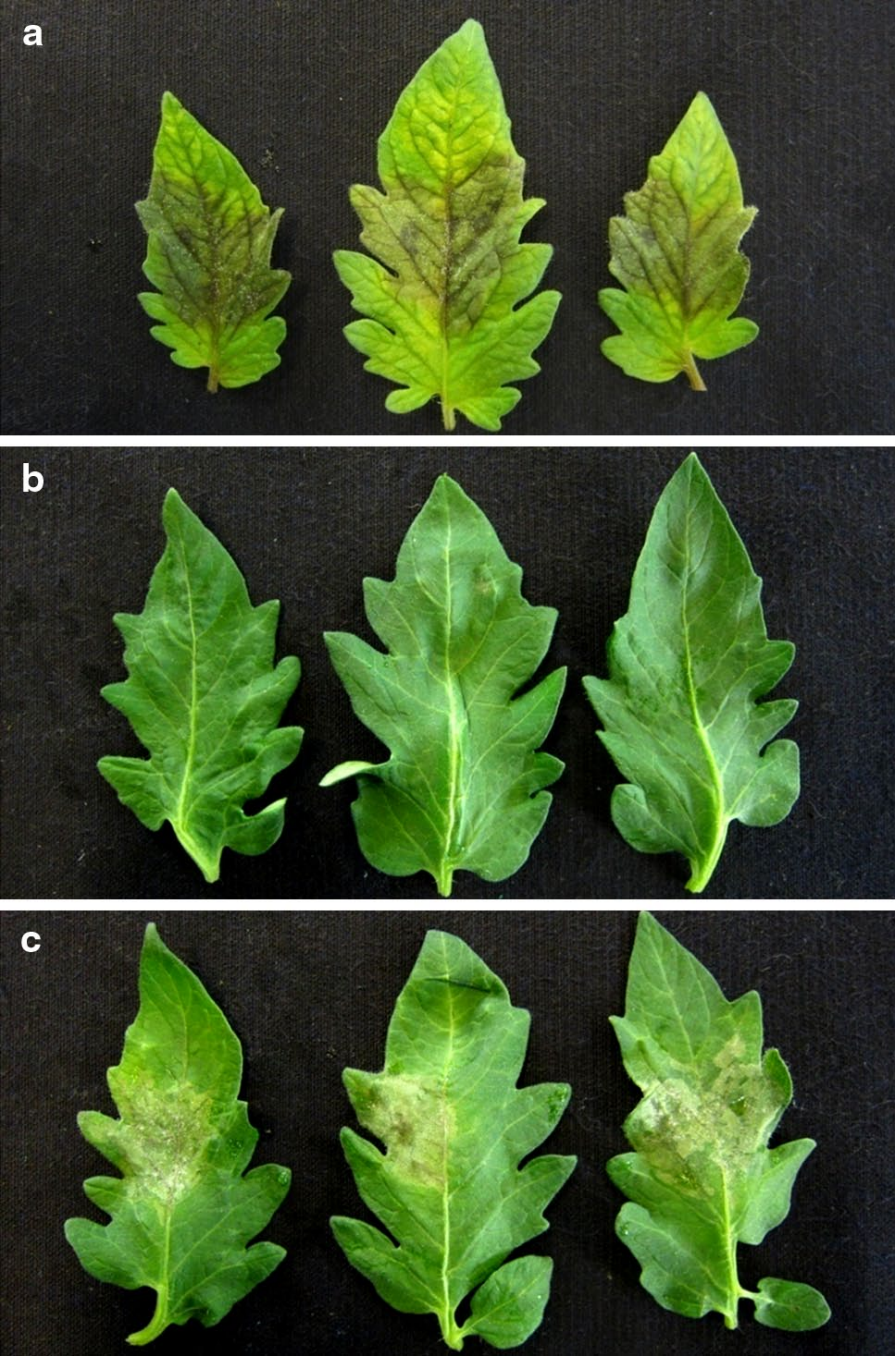
Expression of the resistant allele of *Ph*-*3* in susceptible Moneymaker resulted in resistance to *P. infestans*. Transgenic lines were tested in two independent experiments. **a** Non-transformed Moneymaker showed mycelium growing on the infected leaf areas; **b** transformed Moneymaker expressing *Ph*-*3* showed no symptom and **c** transformed Moneymaker not expressing *Ph*-*3* showed mycelium growing on the infected leaf areas. It is worthwhile to note that non-transformed Moneymaker plants were grown from seeds and that transformed Moneymaker were from cuttings. Three leaflets in **a**, **b** or **c** were taken from one inoculated leaf and three leaves per plant were tested. Photographs were taken 7 days post-inoculation

To analyse the function of the *Ph*-*3* candidate gene, a fragment encompassing 3,565 bp upstream and 1,866 bp downstream of ORF3 was amplified from BAC clone B25E21 and cloned into the binary vector pBINPLUS. The resulting plasmid was used for *Agrobacterium-*mediated transformation of the susceptible tomato cv. Moneymaker. In total, 14 independent transformants containing the *Ph*-*3* gene were obtained and tested for resistance to *P. infestans* isolate T_1,2,4_. Among them, nine transgenic plants were resistant to *P. infestans*, while the remaining five plants were susceptible (Fig. 5; Fig. S2). Compared with CLN2037B, all resistant transgenic plants except CZ-T04 showed comparable or higher expression levels of the *Ph*-*3* gene (Fig. S2). Therefore, the ORF3 under the control of its native promoter and terminator was sufficient to provide resistance to *P. infestans* in the susceptible Moneymaker plants, showing that ORF3 is the *Ph*-*3* gene.

### Structure of *Ph*-*3*

**Fig. 6:**
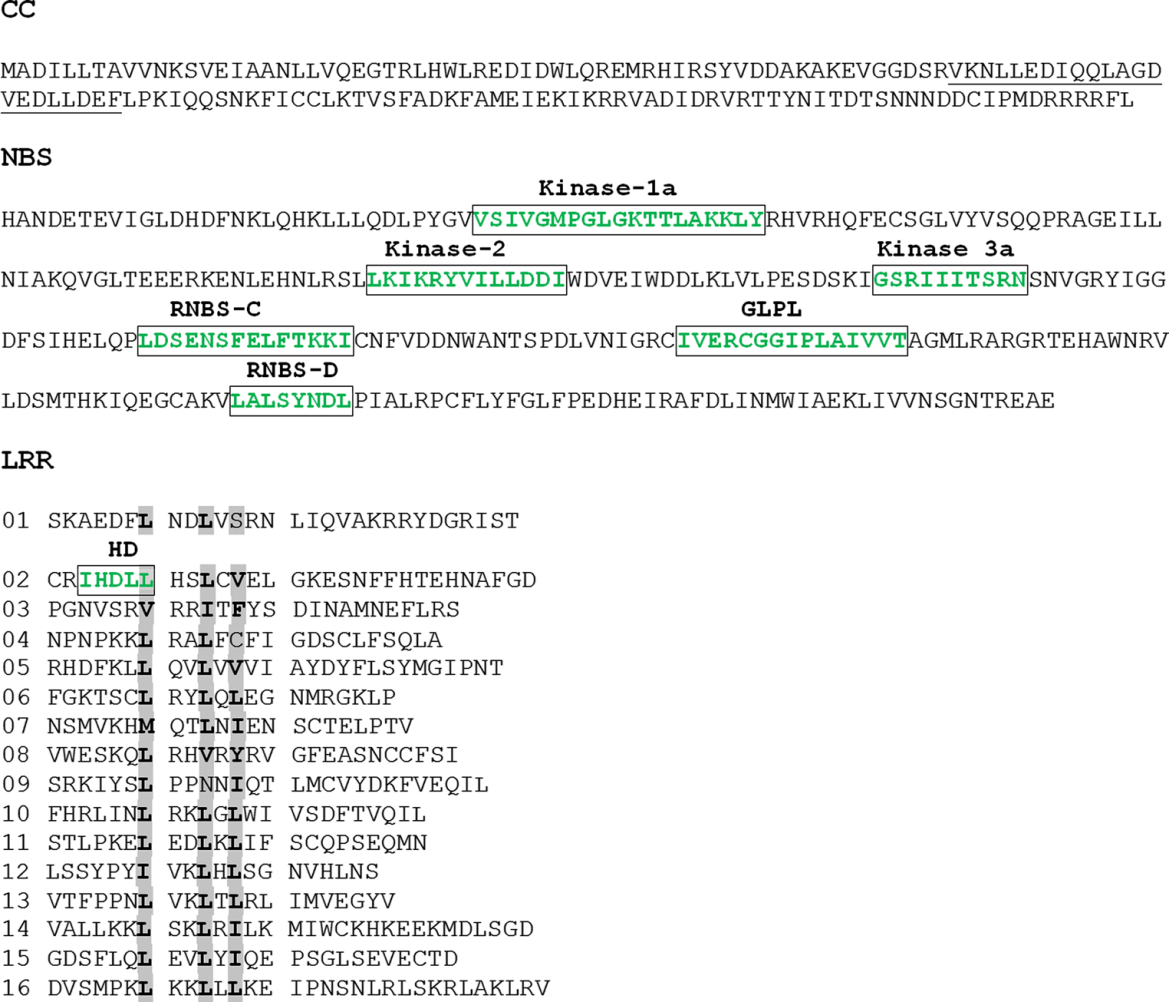
The domain structure of the predicted Ph-3 protein. The predicted coiled coil in the CC domain was *underlined*. *Boxes* indicate positions of conserved NB-ARC motifs. The 16 imperfect LRRs were aligned according to the consensus sequence xxLxLxx (where L represents leucine or other aliphatic amino acid, and x is any residue)

The *Ph*-*3* gene (GenBank accession number: KJ563933) consists of one exon of 2,556 nucleotides, encoding a predicted polypeptide of 851 amino acids (Fig. 6). The deduced Ph-3 protein belongs to the CC-NBS-LRR class of plant R proteins. A predicted coiled-coil (CC) structure is located in the N-terminus between amino acids 63 and 84. Therefore, the entire N-terminus, from amino acid 1 till 150, is referred to as the CC domain. The NBS domain resides between residues 151 and 449, where the conserved NB-ARC motifs are present (van der Biezen and Jones 1998; Meyers et al. 2003). It is remarkable that the HD (H means histidine and D means aspartic acid) domain is located within the predicted LRR region, like the proteins encoded by *R9a* and *Tm-2* ^*2*^ (Jo 2013; Lanfermeijier et al. 2003). The C terminal sequence only loosely fits the consensus for intracellular leucine-rich repeats (LRR), LxxLxxLxLxxC/Nxx (where L represents Leu, Ile, Val or Phe, N stands for Asp, Thr, Ser or Cys, and x is any amino acid) (van Ooijen et al. 2007). However, the consensus sequence for the β-sheet core (xxLxLxx) could be distinguished and totally 16 irregular LRR were found.

**Fig. 7:**
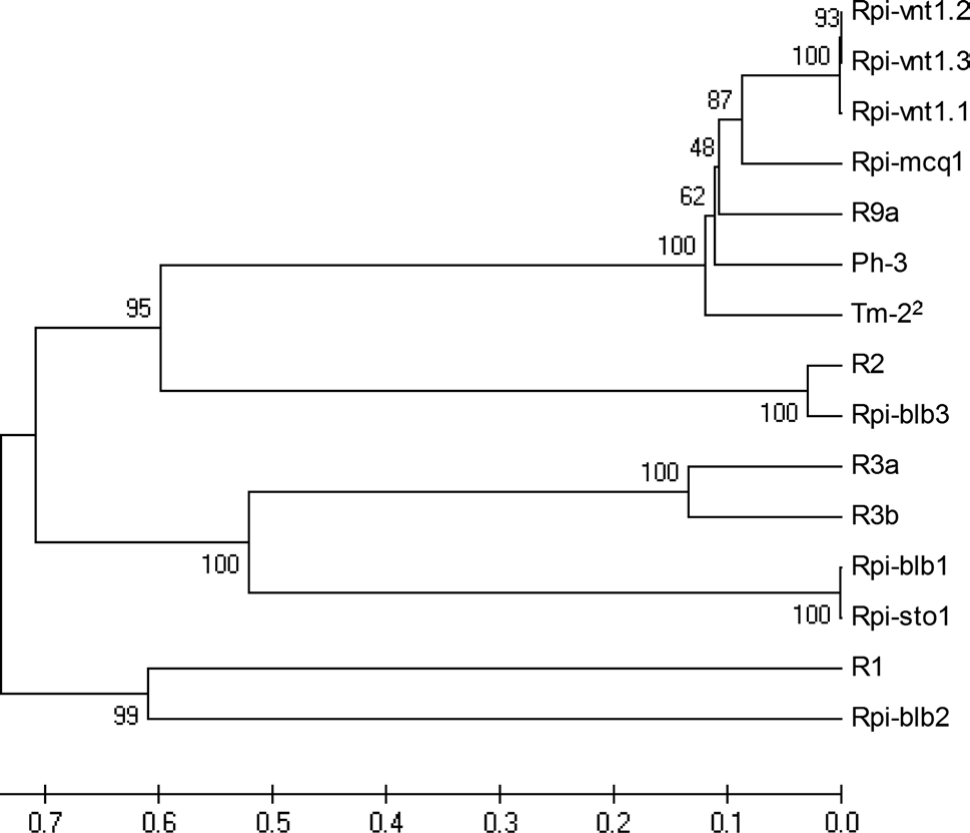
Phylogenetic analysis of *Tm-2* ^*2*^-like resistance proteins and all cloned potato Rpi proteins. The resistance protein sequences were downloaded from GenBank (http://www.ncbi.nlm.nih.gov/genbank/). The phylogenetic tree was performed using MEGA4 with Bootstrap test. *Numbers* at the branches are confidence values

Among the cloned potato *Rpi* genes, *Ph*-*3* shares high identity ranging 74.7–78.7 % to three chromosome-9-derived potato *Rpi* genes, *Rpi*-*vnt1.1* from *S. venturii*, *Rpi*-*mcq1* from *S. mochiquense* and *R9a* from *S. demissum* (Foster et al. 2009; Pel et al. 2009; Jones et al. 2009; Jo 2013) (Table S2). As shown by other studies (e.g. Jupe et al. 2013; Parniske et al. 1997), the lowest identity among these Rpi proteins was found in the LRR domain. Taking *Ph*-*3* and *Rpi*-*vnt1.1* as an example, the identity in LRR domain is 63.3 % while the CC and NBS domains show an identity of 90.7 and 91.4 %, respectively. In addition, *Ph*-*3* also shares high amino acid identity to the tomato mosaic virus resistance gene *Tm-2* ^*2*^ from *S. peruvianum*, which is located near the centromere of chromosome 9 (Table S2) (Lanfermeijier et al. 2003). All chromosome-9-derived proteins (Ph-3, Rpi-vnt1.1, Rpi-vnt1.2, Rpi-vnt1.3, Rpi-mcq1, R9a and Tm-2^2^) belong to one clade (Fig. 7), which is distinct from other identified potato Rpi proteins.

## Discussion

In a previous study, we have demonstrated that *Ph*-*3* belongs to the NBS-LRR *R* gene cluster on chromosome 9 (Zhang et al. 2013). Unfortunately, the full length of candidate *R* gene homologs could not be amplified from the *Ph*-*3*-containing tomato lines with the primers designed according to the Heinz1706 reference genome. The failure in such a homology-based cloning was likely due to SNPs present in sequence of *S. pimpinellifolium* L3708 compared to the Heinz1706 reference genome (Fig. 2). We have therefore taken a map-based cloning approach for cloning of the *Ph*-*3* gene, which is the first cloned tomato late blight *R* gene. Like the most cloned *R* genes, the *Ph*-*3* gene also belongs to the NBS-LRR complex. Compared with the tomato Heinz1706 genome sequence, there is a deletion in the *Ph*-*3* region of *S. pimpinellifolium* L3708 where RGAs are clustered. In Heinz1706, there are four RGAs while in L3708 there are only two RGAs. One of the two RGAs, ORF3, is confirmed to be the *Ph*-*3* gene.

## A hotspot carrying *Rpi* genes on chromosome 9 of *Solanum* species

It is well known that the NBS-LRR class of *R* genes is often clustered in the genome as a result of tandem and segmental duplications (Hulbert et al. 2001; Leister 2004; McDowell and Simon 2006). Occasionally, all *R* genes in one cluster are functional, as is the case for *R3* locus for late blight resistance in potato (Huang et al. 2005; Li et al. 2011b). In this study, however, only one RGA in the *Ph*-*3* cluster contributes to the resistance for late blight. The *Ph*-*3* gene is located at the end of the long arm of chromosome 9, a region carrying many *Rpi* genes in Solanaceae. In *Solanum* species, *Rpi* genes including *Rpi*-*vnt1.1*, *Rpi*-*mcq1*, *R8*, *R9a*, *Rpi*-*edn2* and *Rpi*-*dlc1*, are located in this region (Pel et al. 2009; Smilde et al. 2005; Jo et al. 2011; Jo 2013; Verzaux 2010; Golas et al. 2010). Due to high variability of *R* gene clusters across species and lack of flanking sequences of these *Rpi* genes, it is hard to determine if all or any of these genes are orthologs of *Ph*-*3*. Nevertheless, *Ph*-*3* exhibits highest identity to *Rpi*-*vnt1.1*, *Rpi*-*mcq1* and *R9a*. These Rpi proteins are quite conserved in the CC and NBS domain (Jupe et al. 2013), while there is a high degree of amino acid variability in predicted solvent exposed residues of the LRR parallel β-sheet structure, a determinant of recognition specificity (Parniske et al. 1997).

The changes of generating a gain of function allele by random mutation alone are extremely low (Parniske and Jones 1999). Evolution of *R* genes is driven by gene duplication and unequal crossing-over followed by diversifying selection (Michelmore and Meyers 1998; Hulbert et al. 2001). For example, the presence of tandemly duplicated homologous sequences at the *Cf*-*4*/*Cf*-*9* locus promote chromosome mispairing followed by unequal crossing-over or gene conversion events (Thomas et al. 1997). In the *Ph*-*3* cluster, there are two and four RGAs in *S. pimpinellifolium* L3708 and *S. lycopersicum* Heinz1706, respectively. All RGAs in these two genomes share high identity with each other ranging from 87.8 % (*SlRGA1* and *SlRGA3*) to 97.5 % (*SlRGA3* and *SlRGA4*) (Table S1), which possibly promotes the unequal homologous recombination. Furthermore, we indeed showed that the *Ph*-*3* allele in the recombinant 8-25 resulted from an unequal crossover between the *Ph*-*3* gene and the susceptible allele, which led to a chimeric and non-functional RGA. It suggests that chromosomal rearrangements within *R* gene clusters do occur, resulting in the formation of a novel allele.

## A combined use of tomato and potato *Rpi* gene to achieve durable resistance

Both tomato and potato are hosts of *P. infestans*. The resources of late blight resistance in tomato germplasm are less abundant than in the potato. So far, all tomato *Rpi* genes, which are useful for resistance breeding, are identified in the wild species *S. pimpinellifolium* (Bonde and Murphy 1952; Gallegly and Marvel 1955; Peirce 1971; Moreau et al. 1998; Black et al. 1996a, b; Chunwongse et al. 2002; Foolad et al. 2006, 2008; Merk et al. 2012; Merk and Foolad 2012). Although *Ph*-*3* is widely used in tomato breeding, the resistance of *Ph*-*3* has been overcome. Chunwongse et al. (2002) reported that four isolates were virulent on the *Ph*-*3* donor L3708. Therefore, it is necessary to investigate if other wild relatives of tomato can provide novel monogenic *Rpi* genes conferring race-nonspecific resistance.

Durable disease resistance is the ultimate goal of many breeding programmes. Durable resistance has no particular genetic basis. It is a consequence of both the nature of resistance in the plant and the evolutionary potential of the pathogen (Michelmore et al. 2013). Some monogenic *R* genes, such as *Lr34* in wheat, *mlo* in barley and other species, have proved durable over many years of agricultural use (Krattinger et al. 2013; Jørgensen 1992; Bai et al. 2008). For late blight, however, single *R* genes were quickly overcome in the field. Stacking of two or multiple *Rpi* genes can confer resistance to a broad and complementary set of isolates (Zhu et al. 2012, 2013). For stacking strategy, the knowledge of interaction between *P. infestans* (effectors) and host (*R* genes) is essential, which helps to evaluate the durability of *R* genes (Vleeshouwers et al. 2008). The *Ph*-*3* gene has high identity with two potato *Rpi* genes (*Rpi*-*vnt1.1* and *R9a*) of which corresponding effectors are known (Pel 2010; Jo 2013). Whether *Ph*-*3* recognizes these effectors is still not clear.

An alternative approach to manage late blight in tomato is to introduce potato *Rpi* genes into tomato. It has been reported that the potato *Rpi* genes *Rpi*-*blb1*, *Rpi*-*blb2*, *R1*, *R3a*, *Rpi*-*vnt1.1*, and *Rpi*-*mcq1* were functional in tomato (van der Vossen et al. 2003, 2005; Jia et al. 2009; Foster et al. 2009; Jones et al. 2009). The *Rpi*-*blb1* or *Rpi*-*blb2* transgenic tomato plants not only showed resistance to *P. infestans* isolates from potato, but also to the isolates from tomato (van der Vossen et al. 2003, 2005; Jia et al. 2009), which illustrates the potential effectiveness of the employment of potato *Rpi* genes in tomato. Furthermore, we observed that the tomato line CLN2037B containing *Ph*-*3* were resistant to multiple potato isolates (data not shown), suggesting that *Ph*-*3* could protect potato from late blight. However, Oyarzun et al. (1998) observed a greater specificity of isolates for their first host than for their alternative host. Also Vega-Sánchez et al. (2000) found that tomato and potato were attacked by two separate, host-adapted populations of *P. infestans*. Therefore, it is still unclear that the resistance to potato isolates is mediated by the *Ph*-*3* gene or other host or pathogen factors. In addition, the *Ph*-*3* gene provides only partial resistance (Zhang et al. 2013). Although complete resistance was achieved when the *Ph*-*3* gene was highly expressed under its native promoter, an absolute correlation between gene expression level and resistance was not found (Fig. S2). Compared to CLN2037B, the *Ph*-*3* gene was significantly higher expressed in three transgenic plants (T10, T09, T15) which showed no symptoms on the inoculated leaves. However, not all transgenic plants having a similar level of expression as the one in CLN2037B showed resistance. One possible reason is the difference at insertion locations of the *Ph*-*3* gene. Alternatively, the expression of *Ph*-*3* might be influenced by developmental stages and environments because leaves used for inoculation and RNA extraction were different. Thus, the resistance level in these transgenic tomato lines needs to be confirmed by testing their progenies. Further, transformation of *Ph*-*3* into susceptible potato cultivars and analysis of their resistance level and spectrum will verify the potential effectiveness of the employment of *Ph*-*3* in potato breeding programmes.

## Electronic supplementary material

Below is the link to the electronic supplementary material.

Expression level of ORF2 and ORF3 in leaves of the *Ph*-*3*-contained line CLN2037B. The level of gene expression was normalized against the housekeeping gene *EF1α*. Standard variation bar was calculated with three biological replicates (PNG 29 kb)

The expression levels of the *Ph*-*3* gene and lesion sizes on the leaves of the transgenic plants challenged with *P. infestans*. (a) The expression level of the *Ph*-*3* gene. CLN2037B is a tomato inbred line containing the *Ph*-*3* gene. T01 to T23 were independent primary transformants. The *Ph*-*3* expression level was normalized against the housekeeping gene *EF1α*. Means and standard variations were calculated with three technical replicates from a cDNA pool of three leaves of each primary transgenic plant. (b) The lesion sizes on the leaves post inoculation with *P. infestans*. Grey bars indicate resistant plants, while white bars indicate susceptible plants in both (a) and (b). *indicates significance at 0.05 level (PNG 115 kb)

Supplementary material 3 (DOCX 15 kb)
