# Supplementary figures and images for: The Ph-3 gene from Solanum pimpinellifolium encodes CC-NBS-LRR protein conferring resistance to Phytophthora infestans
Source: Theor Appl Genet. 2014 Apr 23;127(6):1353–64. doi: 10.1007/s00122-014-2303-1 (PMC4035550; doi:10.1007/s00122-014-2303-1)

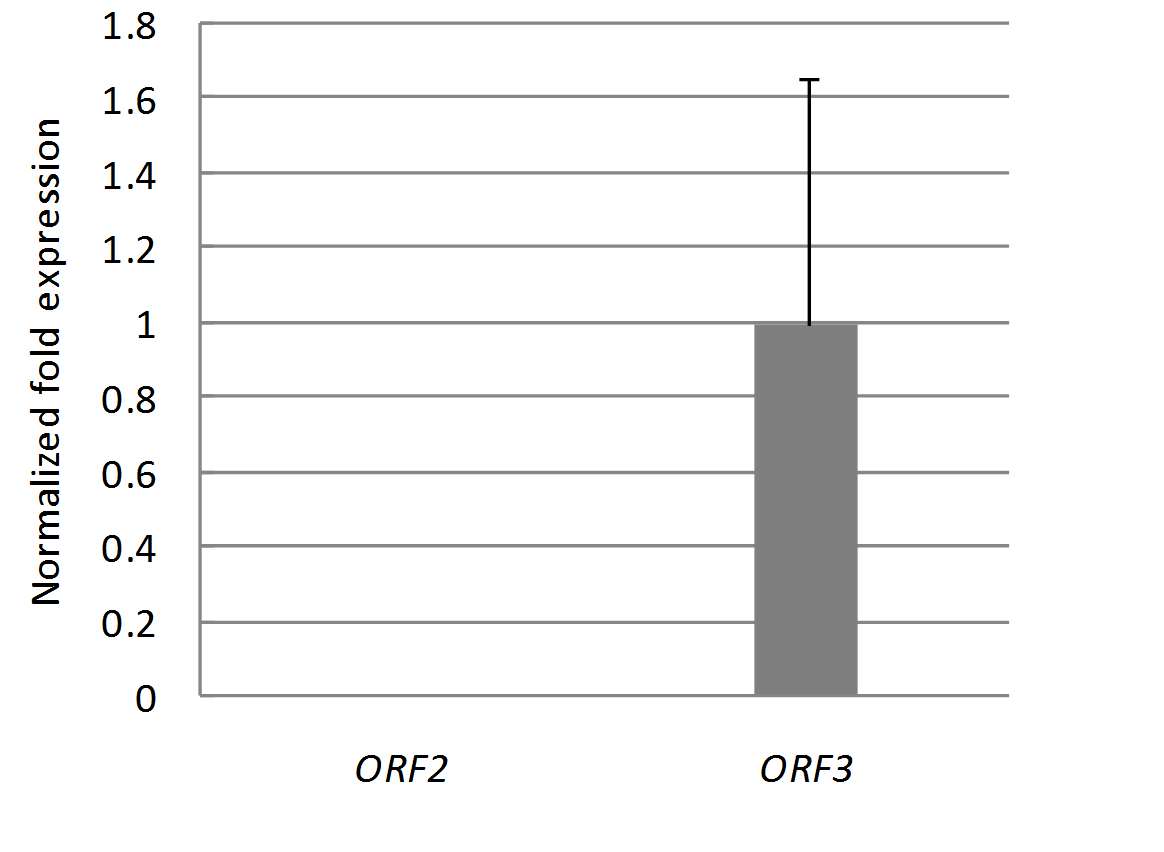

Supplement: Supplementary file 1 — Expression level of ORF2 and ORF3 in leaves of the Ph-3-contained line CLN2037B. The level of gene expression was normalized against the housekeeping gene EF1α. Standard variation bar was calculated with three biological replicates (PNG 29 kb) [file 122_2014_2303_MOESM1_ESM.png]

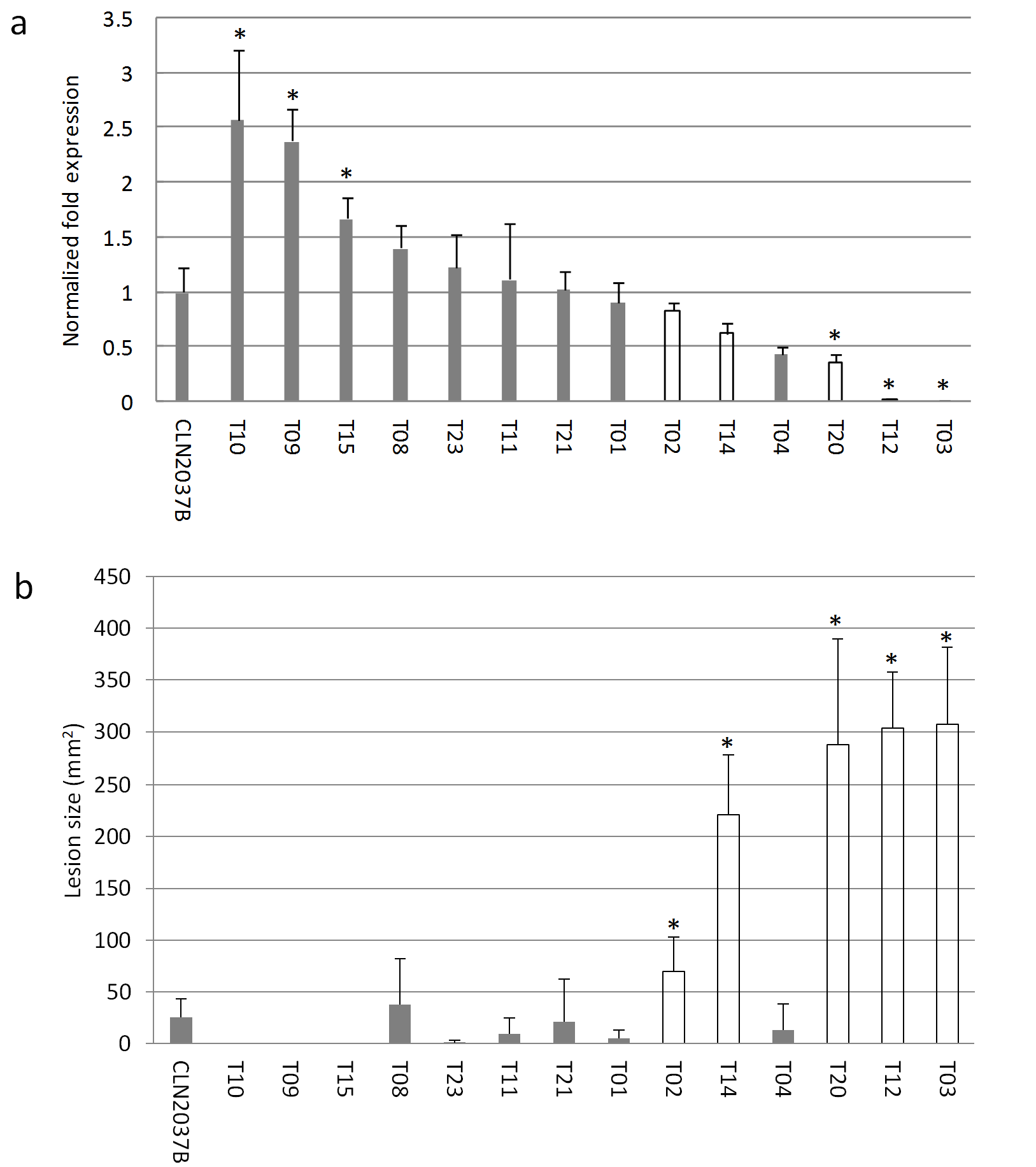

Supplement: Supplementary file 2 — The expression levels of the Ph-3 gene and lesion sizes on the leaves of the transgenic plants challenged with P. infestans. (a) The expression level of the Ph-3 gene. CLN2037B is a tomato inbred line containing the Ph-3 gene. T01 to T23 were independent primary transformants. The Ph-3 expression level was normalized against the housekeeping gene EF1α. Means and standard variations were calculated with three technical replicates from a cDNA pool of three leaves of each primary transgenic plant. (b) The lesion sizes on the leaves post inoculation with P. infestans. Grey bars indicate resistant plants, while white bars indicate susceptible plants in both (a) and (b). *indicates significance at 0.05 level (PNG 115 kb) [file 122_2014_2303_MOESM2_ESM.png]
