# Supplementary material for: The Ph-3 gene from Solanum pimpinellifolium encodes CC-NBS-LRR protein conferring resistance to Phytophthora infestans
Source: Theor Appl Genet. 2014 Apr 23;127(6):1353–64. doi: 10.1007/s00122-014-2303-1 (PMC4035550; doi:10.1007/s00122-014-2303-1)
Supplement: Supplementary file 3 — Supplementary material 3 (DOCX 15 kb) [file 122_2014_2303_MOESM3_ESM.docx]

**Table S1** The nucleotide identity (%) between predicted RGAs at the *Ph-3* locus in L3708 and Heinz1706

|  | *SlRGA2* | *SlRGA3* | *SlRGA4* | *SpRGA1* | *Ph-3* |
| --- | --- | --- | --- | --- | --- |
| *SlRGA1*^a^ | 95.71 | 87.76 | 88.39 | 94.21 | 88.74 |
| *SlRGA2* |  | 87.79 | 88.54 | 95.02 | 88.65 |
| *SlRGA3* |  |  | 97.49 | 88.54 | 95.15 |
| *SlRGA4* |  |  |  | 89.15 | 95.85 |
| *SpRGA1*^b^ |  |  |  |  | 88.74 |

^a^ *SlRGA1-4* refer to RGAs in *S. lycopersicum* Heinz1706. ^b^ *SpRGA1* refers to ORF2 in the BAC clone B25E21.

**Table S2** The amino acid identity (%) between *Ph-3* and other chromosome-9-derived potato *Rpi* genes as well as *Tm-2^2^*

|  | *R9a* | *Rpi-mcq1* | *Rpi-vnt1.1* | *Tm-2^2^* |
| --- | --- | --- | --- | --- |
| *Ph-3* | 74.74 | 78.73 | 77.79 | 73.91 |
| *R9a* |  | 80.74 | 77.4 | 72.59 |
| *Rpi-mcq1* |  |  | 80.97 | 77.82 |
| *Rpi-vnt1.1* |  |  |  | 75.15 |
